# Perl module and PISE wrappers for the integrated analysis of sequence data and SNP features

**DOI:** 10.1186/1756-0500-2-92

**Published:** 2009-05-24

**Authors:** B Jayashree, A BhanuPrakash, Anusha Jami, P Srinivasa Reddy, Spurthi Nayak, Rajeev K Varshney

**Affiliations:** 1 Bioinformatics Unit, International Crops Research Institute for the Semi-Arid Tropics (ICRISAT), Patancheru 502 324, Andhra Pradesh, India; 2 Bioinformatics Centre, University of Hyderabad, Gachibowli, 500046, Hyderabad, Andhra Pradesh, India; 3 Centre of Excellence in Genomics, International Crops Research Institute for the Semi-Arid Tropics (ICRISAT), Patancheru 502 324, Andhra Pradesh, India

## Abstract

**Background:**

There is a need for software scripts and modules for format parsing, data manipulation, statistical analysis and annotation especially for tasks related to marker identification from sequence data and sequence diversity analysis.

**Results:**

Here we present several new Perl scripts and a module for sequence data diversity analysis. To enable the use of these software with other public domain tools, we also make available PISE (Pasteur Institute Software Environment) wrappers for these Perl scripts and module. This enables the user to generate pipelines for automated analysis, since PISE is a web interface generator for bioinformatics programmes.

**Conclusion:**

A new set of modules and scripts for diversity statistic calculation, format parsing and data manipulation are available with PISE wrappers that enable pipelining of these scripts with commonly used contig assembly and sequence feature prediction software, to answer specific sequence diversity related questions.

## Background

Single Nucleotide Polymorphisms (SNPs) are commonly found throughout the genome and provide dense maps over small chromosomal regions. The recent advances in sequencing and genotyping have made large scale SNP diversity analysis possible in several crop species. This helps assess genome variation that can then be harnessed for crop improvement. Sequence diversity information may be desirable across defined groups of sequences, such as candidate gene transcripts from different genotypes, or assembled transcripts for a particular marker from more than one genotype. The grouping could be based on the objective of the study – across race, location, genes or regions within genes. Sequence data analysis usually involves steps such as clustering of sequence data, to determine redundancy levels. Sequence assembly is carried out to generate consensus sequences or contigs and singlets. The user then processes this output to determine presence of microsatellites or SNPs. Along with SNP identification it is also desirable to obtain other aspects from the alignment; such as SNP and *indel *(insertion-deletions) frequency, the type of variant and haplotypes, PIC value for the SNP and haplotype besides nucleotide diversity (π). Validation of predicted SNP(s) through wet lab experiments is the next step to convert the identified SNP into a genetic marker. Although more than 30 SNP genotyping platforms are currently available, these are both expensive and demand considerable expertise. One solution for validating the identified SNP(s) through cost effective SNP genotyping platform is development of CAPS (cleaved amplified polymorphic sequences) marker by predicting the restriction enzyme that can use the identified SNP as a recognition site.

There are several available software solutions for sequence clustering, and a few popular ones for assembly. The popular group of Clustal programmes [1], d2-cluster [2] for EST clustering and cap3 or PCAP [3,4], the TIGR assembler [5] or Phrap [6] are used for sequence assembly. Similarly, there are many freely available software programmes for the identification of SNPs[7-10]. DnaSP reports on nucleotide polymorphism features from aligned sequence data [11]. None of them however automate group wise identification and reporting of polymorphism statistics and more importantly consider the presence of heterozygous loci in the sequence data. Many available programs read heterozygous SNPs as missing/bad quality sequence data and thus do not consider them for analysis. As a result features such as sequence diversity, PIC of SNP and haplotypes, etc. may be underestimated. The need for a module that could report SNP features for any number of user defined groups coupled with the need to be able to calculate statistics taking into consideration the presence of heterozygous loci led to the development of the SNP DIVersity ESTimator module (*divest.pm*).

Sequence analysis involves pipelining of data from one software to another and often also includes branched flows such as when annotation of sequences with putative function is also a requirement. Format conversion scripts to convert output of one program to input of another are needed when the user wants to pipeline several tools and modules. Along with output parsing scripts, some degree of automation can be achieved in data analysis tasks. The availability of software environments for pipelining and workflow management help the user to create custom analysis pipelines.

The PISE programme [12,13] is a robust environment that has been around for many years now and allows integration of internal scripts/tools that are part of the target execution environment as well as external tools that a user runs. Ease of use is achieved through the creation of a graphical user interface (GUI) for all of the programmes/scripts available in the environment and the chaining together of scripts to facilitate automation and analysis. So rather than reinvent a workflow environment; we implemented PISE locally and provided PISE XML wrappers for the Perl scripts and modules developed by us, besides making them available as web services. The availability of the programs and wrapper sripts allow users to implement versatile pipelines either in the familiar browser environment or in the Taverna workbench. The modules and scripts are available to all interested users.

## Implementation

**Figure 1:**
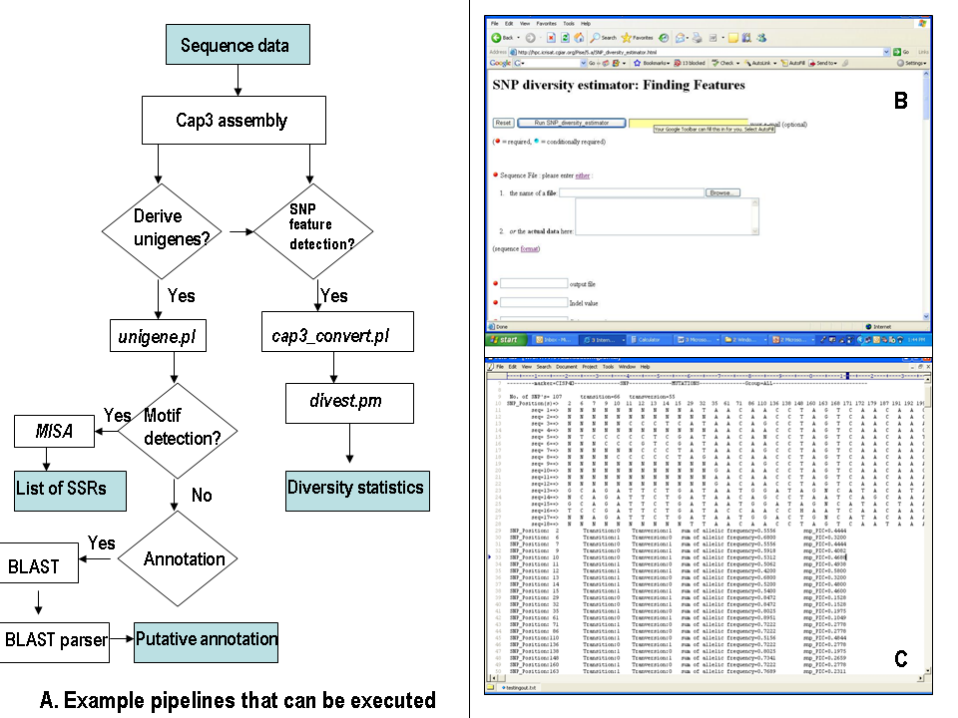
**A. An example pipeline that can be executed through the PISE environment**. Blue boxes represent inputs and outputs in the pipeline. **B**. The PISE interface to the diversity estimator module. **C**. Screenshot of *divest.pm *ouput.

The source code for the programs has been written in Perl 5.8. For the diversity estimator module, Bioperl modules have been used. The scripts have been tested within the PISE environment and are being used independently as well, on machines running either Linux or Windows OS. An example pipeline that makes use of the scripts written is indicated (Figure 1A). The modules allow a large amount of data to be processed; we have tested it to work with up to 99 user defined groups consisting of up to 225 sequences per group. Interface development involves writing the XML specification for the programme/script/module using the grammar provided , where file manipulations and redirection of output files to another programme are also specified. The scripts are also available as a soaplab service and can so be called through the Taverna workflow tool.

## Results and discussion

### Functionality of Perl modules and scripts

The module *divest.pm *is the diversity estimator module that reads an input file derived from assembly programmes like cap3 or the alignment file output from multiple sequence alignment programmes like ClustalW or BioEdit. The file contains the assemblies of reads for each user defined group. The user can choose to calculate diversity statistics for all groups or one particular group. The statistics calculated include nucleotide diversity, number of SNPs, and type of variant whether transition or transversion, SNP frequency, number of haplotypes, the PIC (polymorphism information content) of SNP and haplotype, haplotype frequency and also number and frequency of indels.

SNP and indel frequency are simply calculated as the total length of the sequence in base pairs/number of SNPs or indels. The count of number of haplotypes and haplotype frequency is based on the standard methods [14]. An important addition in these calculations is the consideration of heterozygous loci. The user can replace an N with an H (in case of true heterozygote) based on two peaks in chromatograms obtained from sequencing machines. The algorithm assigns 'H' two alleles (A/T or C/G each with a value of 0.5) and this is used in haplotype analysis and sequence diversity calculations.

The nucleotide diversity π is calculated using the formula [15]



where k is the number of SNPs identified in an alignment of 'n' genotypes, L is the number of basepairs and .

The PIC of SNP is calculated using the formula [16]



where *p*_i _is the frequency of the *i*th allele at a given SNP locus.

The haplotype diversity is calculated using the formula [17,18]



where K is the number of haplotypes, p_i _is the frequency of haplotypes and n is the total number of reads.

The statistics generated is exported in Excel format, besides the script also generates input file to Network 4.502 . Network helps generate evolutionary trees from various data including haplotype and haplotype frequency data.

Scripts that enable the conversion of a multiple sequence alignment into the diversity estimator input file have been currently written for the ClustalW and cap3 programmes. Another script that enables concatenating cap3 contig and singlets file is also available, the output of this file generates a 'unigene' file that can serve as input to the MISA microsatellite analysis programme. An example analysis pipeline is provided in Figure 1 along with the PISE interface to the diversity estimator programme and its output.

### Accessibility of modules and scripts

**Figure 2:**
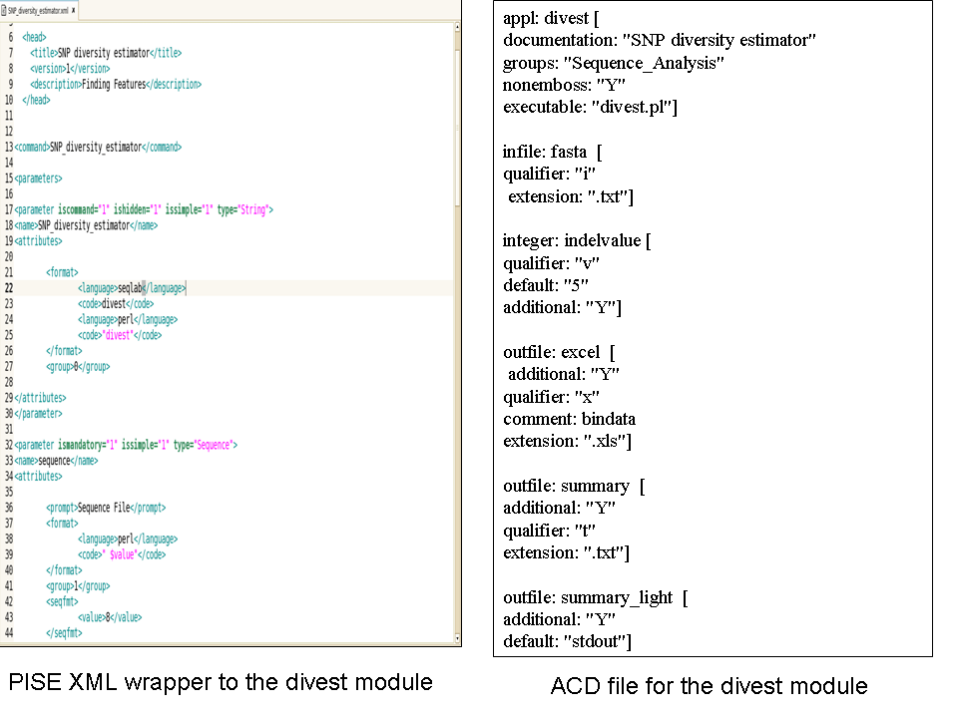
**PISE XML wrapper and ACD file for the diversity estimator module**.

Figure 2 illustrates the XML specification for a module like divest.pm. Chaining together of programmes is made feasible through the parameter setting in the XML file, piping output files to input file of the next programme. In a PISE implementation available at , all mentioned scripts are available under assembly, format parsing, statistics and data manipulation directories. A sample ACD file for the *divest *module is also given, this provides meta-data regarding the input and output files for this module and can be used by soaplab for deploying *divest.pm *as a web service.

### Intended use

The modules and scripts being provided through the PISE environment can be used for various purposes (a) identifying microsatellites in a set of unigenes, (b) calculating the diversity between two or more groups of data based on geographical location of the genotypes (e.g. cultivated, wild, landraces, etc.), regions of the gene (e.g. intronic and exonic regions) being sampled, (c) sequence diversity features in a candidate gene across several genotypes and (d) identifying SNPs and finding out how many of these are convertible to CAPS, (e) functionally annotate sequences. The ability to chain scripts to generate pipelines reduces the file management burden for the user. A part of the pipeline indicated in Figure 1 can also be executed through the Taverna workflow environment [19], which provides web service access besides local service access. The advantage to the user would be the accessibility and integration of databases through web services. Some of the scripts are available as soaplab web services at http://220.227.242.214:8080/soaplab2/; under the heading sequence analysis.

As the modules and scripts are being released as open source code, interested users can continue to use as well as make improvements to them.

## Conclusion

Modules and scripts that facilitate sequence diversity analysis, format parsing and data manipulation are being made available along with source code. The module/scripts can be chained together with other freely available sequence analysis software to generate pipelines for automated data analysis. The pipelining has been currently made feasible through the PISE web interface generator and a part of it can also be executed on the Taverna workbench. The module and scripts are available to interested users.

## Availability & requirements

Modules available from: .

Can be accessed and used at 

Soaplab service: 

Operating System: platform independent

Programming language: Perl

PISE 

SNP2CAPS 

MISA, cap3 (optional; ; )

Any restrictions to use by non-academics: none

## Abbreviations

ACD: Ajax Command Definition; SNP: Single Nucleotide Polymorphism; PIC: Polymorphism Information Content; XML: Extensible Markup Language; PISE: Pasteur Institute Software Environment; CAPS: Cleaved Amplified Polymorphic markers; EST: Expressed Sequence Tag; PCAP: Parallel Contig Assembly Program; CPAN: Comprehensive Perl Archive Network; MISA: MIcroSAtellite Identification tool

## Competing interests

The authors declare that they have no competing interests.

## Authors' contributions

BA, AJ coded for the Perl modules and scripts, PSR and SN used the programmes on their datasets, RKV articulated the use cases, indices for the diversity estimator module while JB developed the pipeline architecture and wrote the manuscript.
